# Characterization of the humoral immune response to the EBV proteome in extranodal NK/T-cell lymphoma

**DOI:** 10.1038/s41598-021-02788-w

**Published:** 2021-12-08

**Authors:** Zhiwei Liu, Yomani D. Sarathkumara, John K. C. Chan, Yok-Lam Kwong, Tai Hing Lam, Dennis Kai Ming Ip, Brian C.-H. Chiu, Jun Xu, Yu-Chieh Su, Carla Proietti, Martha M. Cooper, Kelly J. Yu, Bryan Bassig, Raymond Liang, Wei Hu, Bu-Tian Ji, Anna E. Coghill, Ruth M. Pfeiffer, Allan Hildesheim, Nathaniel Rothman, Denise L. Doolan, Qing Lan

**Affiliations:** 1 Division of Cancer Epidemiology and Genetics, 9609 Medical Center Drive, National Cancer Institute, Rockville, MD 20850 USA; 2 Centre for Molecular Therapeutics, Australian Institute of Tropical Health of Medicine, James Cook University, Cairns, Australia; 3 Department of Pathology, Queen Elizabeth Hospital, Hong Kong, SAR China; 4 Queen Mary Hospital, The University of Hong Kong, Hong Kong, SAR China; 5 School of Public Health, Faculty of Medicine, Li Ka Shing (LKS), The University of Hong Kong, Hong Kong, SAR China; 6 Department of Public Health Sciences, University of Chicago, Chicago, USA; 7 Department of Medicine, School of Medicine, I-Shou University, Kaohsiung, Taiwan; 8 Division of Hematology-Oncology, Department of Internal Medicine, E-Da Hospital, Kaohsiung, Taiwan; 9 Hong Kong Sanatorium & Hospital, Hong Kong, SAR China; 10 Cancer Epidemiology Program, Division of Population Sciences, H. Lee Moffitt Cancer Center and Research Institute, Tampa, FL USA

**Keywords:** Cancer epidemiology, T-cell lymphoma, Infection

## Abstract

Extranodal natural killer/T-cell lymphoma (NKTCL) is an aggressive malignancy that has been etiologically linked to Epstein-Barr virus (EBV) infection, with EBV gene transcripts identified in almost all cases. However, the humoral immune response to EBV in NKTCL patients has not been well characterized. We examined the antibody response to EBV in plasma samples from 51 NKTCL cases and 154 controls from Hong Kong and Taiwan who were part of the multi-center, hospital-based AsiaLymph case–control study. The EBV-directed serological response was characterized using a protein microarray that measured IgG and IgA antibodies against 202 protein sequences representing the entire EBV proteome. We analyzed 157 IgG antibodies and 127 IgA antibodies that fulfilled quality control requirements. Associations between EBV serology and NKTCL status were disproportionately observed for IgG rather than IgA antibodies. Nine anti-EBV IgG responses were significantly elevated in NKTCL cases compared with controls and had ORs_highest vs. lowest tertile_ > 6.0 (Bonferroni-corrected *P*-values < 0.05). Among these nine elevated IgG responses in NKTCL patients, three IgG antibodies (all targeting EBNA3A) are novel and have not been observed for other EBV-associated tumors of B-cell or epithelial origin. IgG antibodies against EBNA1, which have consistently been elevated in other EBV-associated tumors, were not elevated in NKTCL cases. We characterize the antibody response against EBV for patients with NKTCL and identify IgG antibody responses against six distinct EBV proteins. Our findings suggest distinct serologic patterns of this NK/T-cell lymphoma compared with other EBV-associated tumors of B-cell or epithelial origin.

## Introduction

Extranodal natural killer T-cell lymphoma (NKTCL; nasal type) is an aggressive malignancy that has been closely linked to infection with Epstein-Barr virus (EBV)^1^. Nearly all NKTCL is EBV positive, with EBV gene transcripts identified in almost 100% of NKTCL tumors^2, 3^. EBV establishes lifelong latency in B cells in over 90% of adults worldwide but causes cancer in only a small fraction of infected individuals^4^. EBV-associated tumors include a subset of Hodgkin and non-Hodgkin lymphoma, as well as epithelial carcinomas of the nasopharynx and stomach^4^. Like EBV-positive Hodgkin lymphoma (HL) and nasopharyngeal carcinoma (NPC), EBV-infected cells in patients with NKTCL have been observed to express genes of latency I (EBNA1 and EBER1/2) or latency II (LMP1/2A/2B, EBNA1, and EBER1/2)^5, 6^. However, the specific role of EBV in the pathogenesis of NKTCL is still poorly understood.

NK and T cells are typically not permissive of EBV infection and, consequently, EBV is not detected in NK or T cells in the blood of healthy carriers, and is only detected at low frequency in tonsillar NK or T cells^7^. A recent study suggested that EBV can infect mature peripheral T cells via binding of EBV glycoprotein gp350 to the cellular membrane protein CD21,^8^ an established receptor for EBV infection of B-cells. However, EBV’s role in NKTCL compared to B-cell lymphomas may differ following initial infection. It is possible that viral protein production is distinct following infection of T-cells, leading to different immune targets against which infected persons mount an antibody response. Study of the humoral (antibody) responses against EBV in patients with NKTCL, and comparison of these patterns to those observed in other EBV-associated cancers, could shed light on pathogenic mechanisms.

The humoral immune response to EBV in NKTCL patients is poorly characterized, with three case-only studies inclusive of a total of 155 patients reported to date^9–11^. Those studies found suggestive elevations in antibody levels against viral capsid antigen (VCA) and early antigen (EA) but not EBV nuclear antigen (EBNA). That pattern is distinct from that observed in other EBV-related cancers including nasopharyngeal carcinoma (NPC), Burkitt lymphoma (BL), and Hodgkin lymphoma (HL)^12–15^. In-depth, comprehensive characterization of serologic profiles that associate with NKTCL, and noting those that are distinct from other EBV-related cancers, could provide insight into the role of specific EBV proteins in the etiology of NKTCL. To investigate this, we utilized a multiplex technology targeting antibody responses to 202 peptide sequences representing the entire EBV proteome to comprehensively evaluate patterns of anti-EBV antibody responses in 205 adults from Hong Kong and Taiwan, including 51 NKTCL cases and 154 matched controls.

## Results

Table 1 shows the distributions of demographic characteristic in 51 NKTCL cases and 154 matched controls from Hong Kong and Taiwan. Cases and controls had a similar sex, age, and study region distribution, reflective of the matched study design. Approximately two thirds of adults recruited were male, and 78.4% of cases (40/51) were recruited in Hong Kong.

**Table 1:** Characteristics of study population, by NK-T cell lymphoma (NKTCL) status in Hong Kong and Taiwan.

Characteristics	NKTCL cases (N = 51)	Controls (N = 154)
**Sex**		
Male	34 (66.7)	102 (66.2)
Female	17 (33.3)	52 (33.8)
**Age at diagnosis/selection (years)**		
18–39	12 (23.5)	34 (22.1)
40–49	13 (25.5)	42 (27.3)
50–59	11 (21.6)	33 (21.4)
60–80	15 (29.4)	45 (29.2)
**Region**		
Hong Kong	40 (78.4)	123 (79.9)
Taiwan	11 (21.6)	31 (20.1)

**Figure 1:**
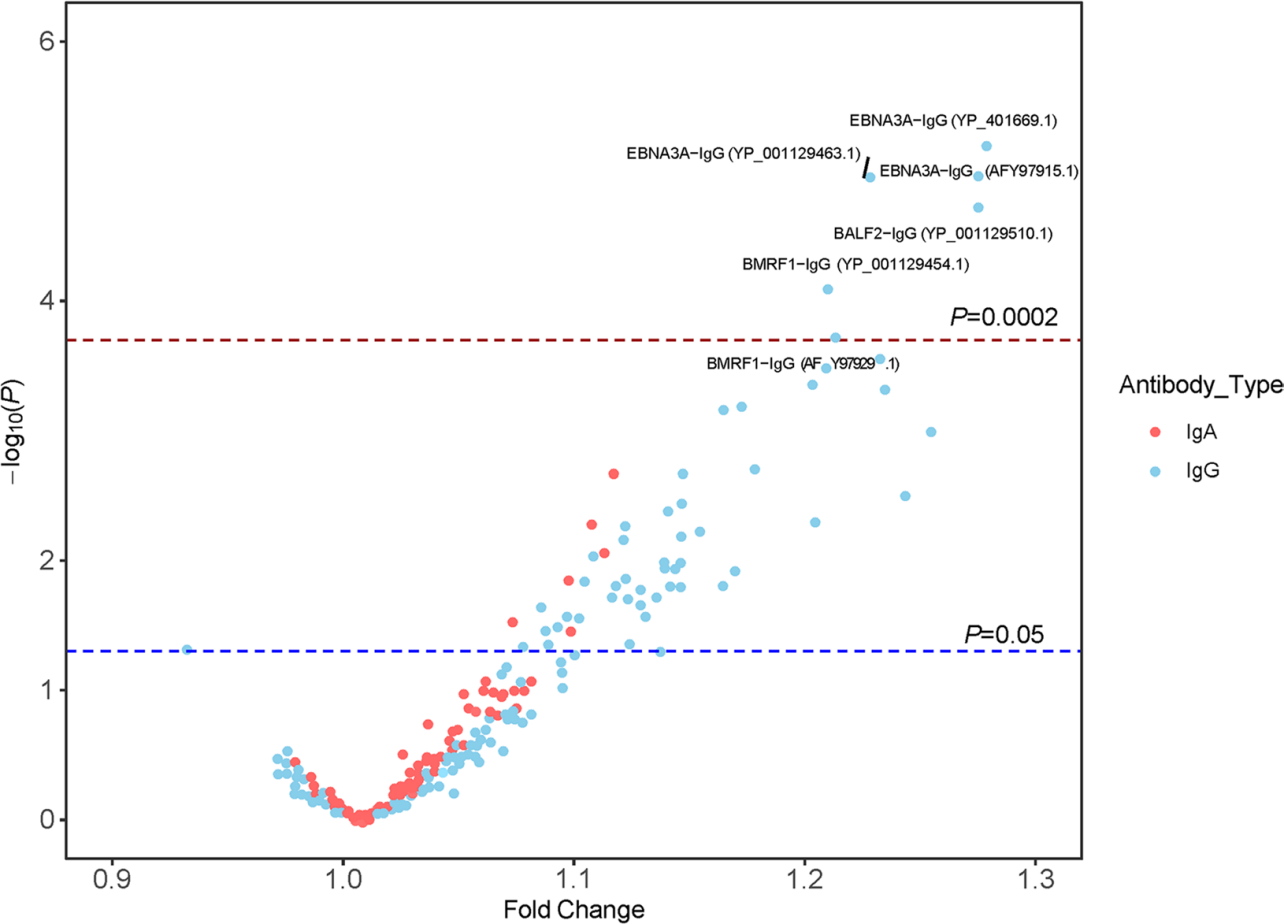
Case–control differences in the mean antibody response for 51 NK/T-cell lymphoma (NKTCL) cases versus 154 controls collected in Hong Kong and Taiwan. The x-axis displays the fold change (case vs. control ratio of standardized signal intensity) for all antibodies with CV ≤ 20%. The y-axis illustrates the p value corresponding to the t-test for a difference in standardized signal intensity between cases and controls. Six IgG-antibodies but no IgA antibodies were significantly elevated in NKTCL cases compared to controls at the *P* < 0.0002 (Bonferroni-corrected *P* < 0.05) threshold.

NKTCL associations were disproportionately observed for IgG rather than IgA antibodies. Case–control comparisons of the mean standardized signal intensity for the 157 IgG and 127 IgA antibodies on the array revealed nominal (*P* < 0.05) elevations in 52 IgG antibodies but only six IgA antibodies (Fig. 1). Six anti-EBV IgG antibodies were significantly elevated in NKTCL cases compared to controls after adjustment for multiple testing (*P* < 0.0002; Fig. 1). Results from the remaining 46 anti-EBV IgG and six anti-EBV IgA antibodies that were nominally significantly elevated in NKTCL cases compared to controls (*P* < 0.05) are shown in Supplementary Table 1. Of note, we did not observe differences in anti-EBV EBNA1 IgG responses between NKTCL cases and controls (Supplementary Table 2).

**Figure 2:**
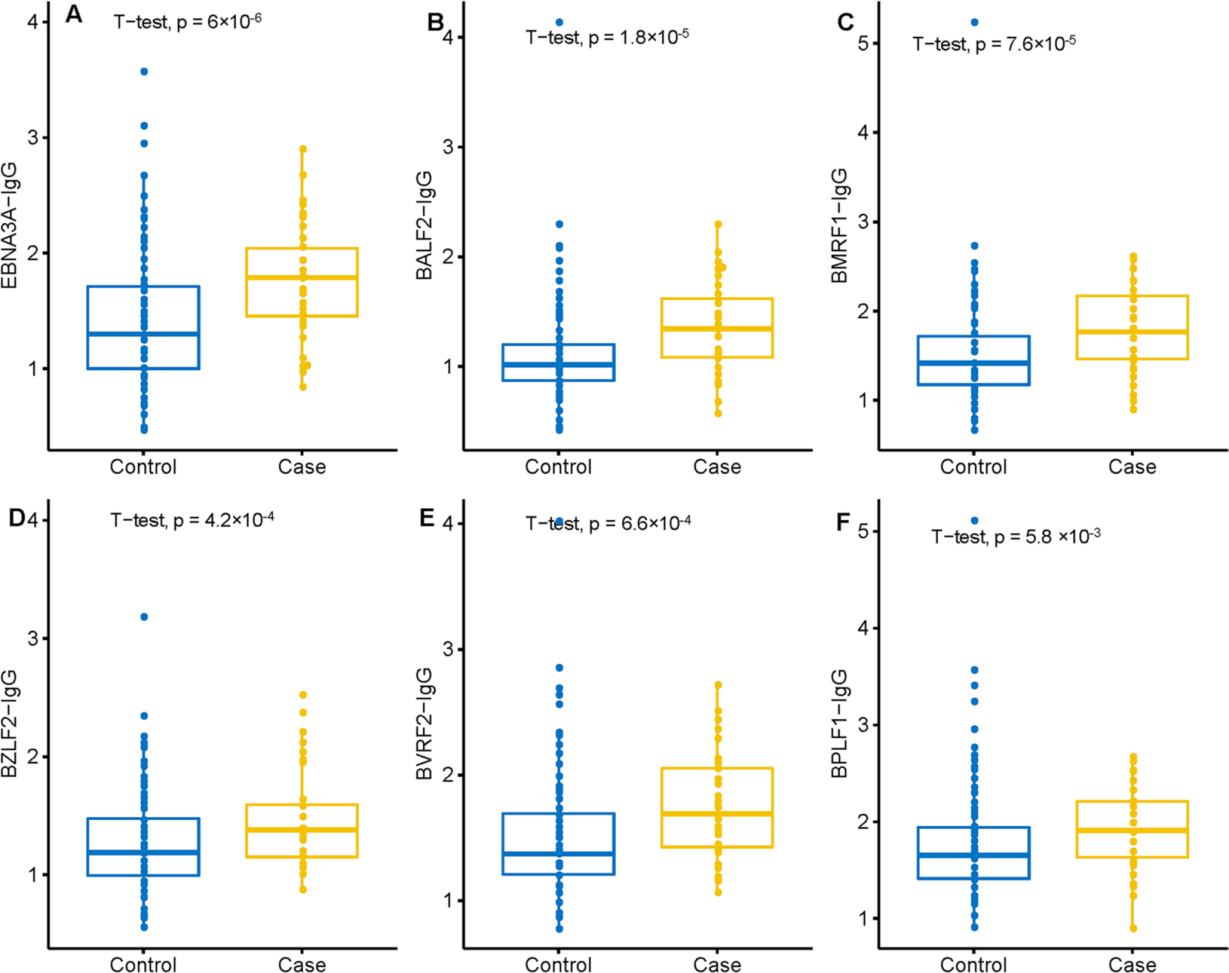
Signal intensity for the six significant anti-EBV IgG antibodies between NK/T-cell lymphoma (NKTCL) and controls, for (**A**) EBNA3A-IgG, (**B**) BALF2-IgG, (**C**) BMRF1-IgG, (**D**) BZLF2-IgG, (**E**) BVRF2-IgG, and (**F**) BPLF1-IgG. *P* values from the t-test are listed.

Using logistic regression models with adjustment for sex, age, and study region, in addition to the six significant anti-EBV IgG antibodies mentioned above, we identified ab additional three IgG antibodies that were significantly elevated in NKTCL cases compared with controls. These elevations had at least a sixfold risk (OR_highest vs. lowest tertile_ ≥ 6.0, Table 2). The most significant *P* value was observed for IgG antibody against latent protein EBNA3A (one of three variants shown in Fig. 2A). Accordingly, the strongest OR effect was observed for antibody against sequences representing EBNA3A (adjusted OR_highest vs. lowest tertile_ = 16.33, 95% confidence interval [CI]: 3.71 to 71.91, *P*-trend = 1.6 × 10^−5^), a protein expressed in latency IIb and III phases that has not been found to be strongly associated with other EBV-associated tumors of B-cell or epithelial origin^12–14^. Pronounced *P* values were also observed for the early lytic proteins BALF2 [EA(D)p138] (one representative variant shown in Fig. 2B) and BMRF1 [EA(D)p47 (one of two variants shown in Fig. 2C). Other IgG antibodies significantly and markedly elevated in NKTCL patients included those targeting antigens representing immediate early and late lytic proteins, BZLF1 [Zebra (Zta)], BVRF2 [VCAp40] and BPLF1 [Tegument protein] (Table 2 and Fig. 2D-2F).

**Table 2:** OR and 95% CI for the association between anti-EBV antibody level and NK-T cell lymphoma (NKTCL) in Hong Kong and Taiwan^a^.

EBV protein and array sequence	Antibody type	t test *P*	NKTCL mean (SD)	Control mean (SD)	Fold change	NKTCL positivity	Control positivity	OR tertile 2 (95% CI)^b^	OR tertile 3 (95% CI)^b^	*P*-trend^c^
**EBNA3A** (YP_401669.1–80,382-82,877)	IgG	5.99 × 10^−6^	1.76 (0.47)	1.38 (0.54)	1.27	96.1%	75.3%	2.44 (0.80–7.45)	6.59 (2.38–18.22)	6.51 × 10^−5^
**EBNA3A** (AFY97915.1–80,252-82,747)	IgG	1.06 × 10^−5^	1.68 (0.46)	1.32 (0.52)	1.27	94.1%	64.3%	4.79 (1.29–17.73)	11.14 (3.21–38.72)	1.84 × 10^−5^
**EBNA3A** (YP_001129463.1–80,447-82,888)	IgG	1.08 × 10^−5^	1.85 (0.44)	1.51 (0.48)	1.22	98.0%	88.3%	8.48 (1.83–39.22)	16.33 (3.71–71.91)	1.63 × 10^−5^
**BALF2 [EA(D)_p138]** (YP_001129510.1–165,796-162,410–1)	IgG	1.79 × 10^−5^	1.37 (0.39)	1.08 (0.41)	1.27	80.4%	52.6%	2.34 (0.75–7.28)	7.29 (2.60–20.43)	3.03 × 10^−5^
**BMRF1 [EA(D)_p47]** (YP_001129454.1–67,745-68,959)	IgG	7.64 × 10^−5^	1.78 (0.44)	1.48 (0.49)	1.20	96.1%	92.9%	2.52 (0.82–7.76)	6.83 (2.45–19.08)	5.70 × 10^−5^
**BMRF1 [EA(D)_p47]** (AFY97929.1–67,486-68,700)	IgG	1.81 × 10^−4^	1.67 (0.45)	1.38 (0.47)	1.21	94.1%	84.4%	2.88 (0.96–8.62)	6.32 (2.27–17.61)	1.60 × 10^−4^
**BZLF1 [Zebra (Zta)]** (YP_001129467.1–91,697-91,197)	IgG	4.19 × 10^−4^	1.49 (0.42)	1.24 (0.39)	1.20	96.1%	74.7%	4.85 (1.3–18.09)	11.13 (3.19–38.78)	1.99 × 10^−5^
**BVRF2 [VCAp40]** (YP_001129501.1–136,465-138,282)	IgG	6.64 × 10^−4^	1.74 (0.40)	1.50 (0.46)	1.17	100.0%	95.5%	2.92 (0.97–8.79)	6.75 (2.39–19.03)	1.19 × 10^−4^
**BPLF1 [Tegument protein]** (CAA24839.1–71,527-62,078–2)	IgG	5.82 × 10^−3^	1.93 (0.40)	1.73 (0.53)	1.11	98.0%	98.7%	2.20 (0.75–6.42)	6.00 (2.25–16.01)	1.17 × 10^−4^

Bold text is used to highlight the canonical EBV protein name. The remaining (non-bolded) text describes the sequence details of the array probe.

CI, confidence interval. SD, standard deviation.

^a^The table is ordered by t test *P* value (lowest to highest).

^b^The odds of being a NKTCL case were calculated from a logistic regression model that included age group (18–39, 40–49, 50–59, 60–80 years), sex, region, and a three-level variable (tertiles) for anti-EBV antibody level. The tertiles were calculated using the underlying antibody distribution among disease-free controls. All ORs are expressed relative to the referent group of tertile 1 (lowest third of antibody distribution).

^c^Two-sided *P* values for trend across marker categories were assessed with the Wald test using categorical values of the proteins with 1 degree of freedom.

We next examined the correlations between these nine highly differentially expressed anti-EBV IgG antibodies. Strong correlations were observed for antibodies targeting the same antigens (i.e., three variants for EBNA3A and two variants for BMRF1), with correlations ranging from 0.903 to 0.966. More modest correlation was observed between antibodies targeting different antigens, with correlations ranging from 0.313 to 0.747 (Supplementary Fig. 1). In a logistic regression model excluding 3 IgG antibodies (two antibodies against EBNA3A and one against BMRF1) that were highly correlated with antibodies targeting the same antigens, IgG antibodies against EBNA3A, BALF2, and BPLF1 retained statistical significance (*P* < 0.05).

In the sPLS-DA analysis, the top 10 anti-EBV IgG antibodies that were most informative for classifying NKTCL status were those targeting EBNA3A, BALF2, BRLF1, thymidine kinase (TK), BMRF1, and BZLF1 (Supplementary Fig. 2), largely consistent with the most significant antibodies defined using the t-test.

Results from ELISA assays confirmed our array-based findings. For example, we observed that VCA-IgG was significantly elevated among NKTCL cases compared with controls (*P* = 5.2 × 10^−8^, Supplementary Fig. 3A). There was suggestive evidence that VCA-IgA was also elevated (*P* = 0.003) but that association was not statistically significant after adjustment for multiple testing (Supplementary Fig. 3B). Antibodies against EBNA1 (both IgG and IgA) measured by ELISA were not elevated among NKTCL cases compared with controls (Supplementary Fig. 3C-D).

Finally, as an exploratory analysis, we leveraged genotyping data^16^ from 94 controls included in the present study and observed that SNP rs9271588 (which maps to *HLA-DRB1*) was suggestively correlated with the most differentially expressed EBV-antibody EBNA3A-IgG (*P* = 0.06).

## Discussion

This is, to our knowledge, the first study to comprehensively evaluate EBV-directed immunity in adults diagnosed with NKTCL in Asia. We investigated both IgG and IgA responses to each protein expressed in the EBV proteome. Profound differences in the anti-EBV antibody profile between NTCKL patients and matched controls were demonstrated, with significantly elevated IgG antibody responses against six distinct EBV proteins. Notably, the strongest NKTCL–EBV associations mapped to sequences representing EBNA3A (but not EBNA1), suggesting a possible role of this latent protein in disease pathogenesis.

In addition to IgG, we examined anti-EBV IgA antibodies in the context of NKTCL. IgA reflects recent exposure along mucosal surfaces such as the oral epithelium and has proven to be an informative biomarker for EBV-associated epithelial tumors (*e.g.*, nasopharyngeal carcinoma)^12, 17^. However, IgA responses did not significantly differ between NKTCL patients and controls after correction for multiple testing. Although false negative findings cannot be entirely ruled out due to a modest sample size and relatively low activity of IgA antibodies, our findings may indicate that chronic reactivation or recent exposure to the virus at a mucosal site is less important in the pathogenesis of NKTCL.

The unique association of NKTCL with IgG antibodies against EBNA3A has not been previously reported^9, 11, 18^. Coghill et al. have reported an association between IgG antibodies against EBNA3A and BL in Africa, but the magnitude of association is smaller than the present study (OR_highest vs. lowest tertile_ = 1.99)^13^. Although EBV-encoded transcripts and proteins have been detected in patients with NKTCL^1, 6, 19–21^, that expression pattern has generally been consistent with latency I or II infection, which is characterized by expression of EBNA-1, LMP-1, and LMP-2 genes but no other EBNA genes^1, 5, 6, 20, 21^. Therefore, the higher IgG antibody levels against EBNA3A observed in the current study might not be explained by high expression of EBNA3A gene in the tumor tissue. Instead, this observation could reflect a long-term systematic exposure to the upregulation of EBNA3A gene within circulating B cells infected with EBV, which could be an early event during the development of NKTCL.

In agreement with previously reported case-only studies that included 155 patients from the U.S. and China^9, 11, 18^, we confirm elevations in NKTCL patients for IgG antibodies against sequences representing EBV EA, including EAD-p47 and -p138, EBV viral capsid BVRF2 (VCAp40), as well as virion production BPLF1 (tegument protein). Furthermore, in agreement with other epidemiological research^9–11^, we report here elevated IgG antibodies against VCA and EA in patients with NKTCL, but no NKTCL associations with EBNA1. We also expand findings to the switch protein Zta (BZLF1), which has been associated with other EBV-associated malignancies ^12–14^ but not previously studied in the context of NKCTL. It is plausible that, again, systematic exposure to EBV, as indicated by elevations in IgG antibodies against EBV lytic proteins, potentially reflects an impaired T-cell response that allows virus to continue replication and spread from the B-cell compartment to NK/T-cells.

GWAS studies have implicated genetic susceptibility in NTCKL etiology, with signals consistently mapped to the HLA genes in the class II region^16, 22^. In our study, we observed indicative evidence that genetic variation within the HLA class II region affected anti-EBV serologic immunity in controls. It is plausible that people with susceptible HLA variants might mount altered responses to EBV infection that predisposes to NKTCL development^23–26^. Future consortia-based efforts focusing on host genetic variants and anti-EBV antibodies would be required to explore the potential synergistic effects of HLA and EBV in the etiology of NKTCL.

Our results should be interpreted in light of certain methodologic limitations. First, our observations are based on data obtained from a case–control designed study so we are unable to determine whether alterations in anti-EBV antibody responses occurred prior to disease onset; i.e., predisposition to disease, as we have previously reported for other EBV-related tumors using the same EBV antibody array^12–14^. However, the difficulty of conducting an adequately powered prospective study for this rare disease makes it unlikely that this limitation will be easily overcome in the future^3^. Second, this is the only study to date examining the association between the proteome-wide anti-EBV antibody response and NKTCL, and we therefore lack an independent, external dataset for replication. Finally, this array was not designed to detect antibodies to conformational epitopes, which precluded us from examining NKTCL associations for selected transcripts that require glycosylation or other post-transcriptional modifications.

In conclusion, we characterize the antibody response against EBV for patients with NKTCL. Our findings suggest distinct serologic patterns of this NK/T-cell lymphoma compared with other EBV-associated tumors of B-cell or epithelial origin. This NKTCL–specific signature included pronounced differences in the immune response against six viral proteins involved in both latency and replication.

## Methods

### Study population

Plasma samples from 51 NKTCL cases and 154 control adults collected as part of the AsiaLymph, a multi-center hospital-based case–control study in Hong Kong and Taiwan conducted between 2012 and 2017, were selected for study. Eligible cases were aged between 18 and 79 years at diagnosis and living in the geographic region served by the partnering hospital at the time of cancer diagnosis. Cases with a prior history of lymphoma were ineligible. Blood and buccal cell collection were performed at the time of diagnosis and before receiving cancer therapy. Controls were drawn from patients seen at the same partnering hospital for diseases/conditions that were not associated with risk factors under study, including injuries and selected diseases of the circulatory, digestive, genitourinary, and central nervous system. Patients with a history of any lymphoma were not eligible for controls. Of all controls recruited in the two regions (N = 1496; 1119 from Hong Kong and 377 from Taiwan), we randomly selected 154 subjects who were frequency-matched to cases on sex, age (+ /- 5 years), date of enrollment (within 3 months), and region (Hong Kong/Taiwan).

The study was approved by the institutional review boards at each participating site, and the US National Institutes of Health and US National Cancer Institute. Written informed consent was obtained from all participants. All laboratory testing was conducted under a protocol approved by James Cook University Human Research Ethics Committee. All methods were performed in accordance with the Declarations of Helsinki.

### EBV protein microarray

The comprehensive EBV protein microarray chip used in this study has been described in detail previously^12, 27^. Briefly, this microarray contains 202 protein sequences representing almost the entire EBV proteome, including 199 EBV protein sequences generated from five different EBV strains (AG876, Akata, B95-8, Mutu, and Raji) and three synthetic EBV peptides for which circulating antibodies are putative cancer biomarkers (VCAp18, EBNA1, and EAd p47). The 202 sequences represent each of the known open reading frames for EBV, as well as predicted splice variants of those open reading frames. Each of the protein sequences were cloned into the pXT7 expression vector, expressed using the *E. coli* cell-free protein system, and printed onto the microarray. Sequences include N-terminal 10 × histidine (His) and C-terminal hemagglutinin (HA) tags for quality control and to confirm expression on the microarray. High coverage was achieved across the five prototypical EBV strains and ten Chinese strains, with > 97% of the predicted sequences from each strain represented on the microarray at > 99% homology. Four “noDNA” (no translated protein) spots were included to assess person-specific background.

Plasma samples from each of the study participants were tested on this EBV protein microarray as described previously^28^. Slides were scanned on an Axon GenePix 4300B (Molecular Devices, Australia); raw fluorescence intensities were corrected for spot-specific background; corrected data were transformed using variance stabilizing normalization (vsn) in Gmine^29^; and output was standardized to person-specific background (mean ± 1.5 SD of the four “no DNA” spots). Positivity was defined as a standardized signal intensity > 1.0. The standardized signal intensity for each spot was further grouped into three categories, with cutoffs for the categories defined using tertiles of the antibody distribution among the 154 controls.

Thirty-five samples were tested in duplicate, blinded to laboratory personnel, in order to assess assay reproducibility specific to this study population. The average coefficient of variation (CV) across the 202 EBV sequences was 16% [interquartile range (IQR), 14%–20%] for IgG antibody response and 19% (IQR, 16%–22%) for IgA antibody response, demonstrating a good reproducibly of our assay. We excluded 45 IgG and 75 IgA that had CVs > 20%, leaving a total of 157 IgG and 127 IgA antibodies for further analysis.

### Antibody testing using ELISA kits

To internally validate the serological findings from the EBV microarray for putative cancer biomarkers, we utilized commercial ELISA assays to test for IgG and IgA antibodies against recombinant VCA and EBNA1; these two antigens have been extensively investigated in other EBV-related cancers^30^. ELISA assays were purchased from EUROIMMUN, Lübeck, Germany (IgG/IgA antibodies against VCA and IgG antibodies against EBNA1) and Zhongshan Biotech, Zhongshan, China (IgA antibodies against EBNA1)^31, 32^. All samples were tested according to the manufacturers’ instructions. Levels of antibodies were assessed by optical density (OD) values. Reference ODs (rODs) were obtained according to the manufacturers’ instructions by dividing OD values by a reference control. The same thirty-five blinded duplicates tested by microarray were also tested by ELISA to assess assay reproducibility. The CVs for IgG antibodies against VCA and EBNA1 were 6.9% and 7.7%, respectively; for IgA antibodies, CVs against VCA and EBNA1 were 19.1% and 25.1%, respectively.

### Statistical analysis

Differences in the mean standardized signal intensity between NKTCL patients and controls were assessed using an unpaired Student t test. Case–control differences were considered statistically significant at the *P* < 0.0002 threshold (equivalent to Bonferroni-corrected *P* < 0.05) to account for the number of comparisons. Odds ratios (ORs) quantifying the association between the three-level categorical variable for each antibody and NKTCL status were estimated using logistic regression models adjusted for sex, age group (18–39, 40–49, 50–59, 60–80 years), and region. In previous work, no sociodemographic or environmental factors were found to strongly and consistently correlate with elevated anti-EBV antibody responses other than smoking^27, 33, 34^; however, smoking was not associated with NKTCL in a previous study^35^ and therefore was not included in our regression models. *P*-trend values were calculated from a model with each three-level antibody marker treated as an ordinal variable. Antibodies with *P*-trend < 0.0002 threshold (equivalent to Bonferroni-corrected *P* < 0.05) were considered as statistical significance. For results from the ELISA assays, differences in the mean rOD between NKTCL patients and controls were assessed using an unpaired Student t test.

To identify the anti-EBV IgG antibodies that are most informative for distinguishing NKTL cases from controls, we employed sparse Partial Least Squares Discriminant Analysis (sPLS-DA), which was implemented using the splda function in MixOmics R package^36, 37^. The sPLS-DA is a method for identifying the key variables of complex and sparse omics datasets that are associated with a biological outcome of interest and it has been shown to be successful with applications where the number of features far outnumber the number of samples^38^ This procedure involves dimension reduction using Partial Least Squares regression (PLS) for discriminant analysis in combination with a Lasso penalization for feature selection. The number of features selected per component was optimized using tenfold cross validation repeated 5 times and the number associated with the lowest classification error rate was chosen for the final model. The final model was then applied to the entire dataset to obtain the most important anti-EBV IgG antibodies in distinguishing NKTCL cases from controls.

Amongst controls, we estimated the correlation between antibodies using Spearman correlation coefficients. We also evaluated whether previously reported NKTCL-associated genetic variants (i.e., rs13015714, mapped to *IL18RAP*, and rs9271588, mapped to *HLA-DRB1*)^16^ were associated with the level of anti-EBV antibody response using linear regression models adjusted for sex, age group (18–39, 40–49, 50–59, 60–80 years), and region.

## Supplementary Information

Supplementary Information 1.

Supplementary Information 2.

## Data Availability

For original data, please contact zhiwei.liu@nih.gov. Deidentified participant data can be shared.

## Abbreviations

BL: Burkitt lymphoma
CI: Confidence interval
CV: Coefficient of variation
EA: Early antigen
EBNA: Epstein-Barr nuclear antigen
EBV: Epstein-Barr virus
NKTCL: Extranodal natural killer/T-cell lymphoma
IQR: Interquartile range
HL: Hodgkin lymphoma
NPC: Nasopharyngeal carcinoma
OD: Optical density
sPLS-DA: Partial Least Squares Discriminant Analysis
VCA: Viral capsid antigen
