# Supplementary material for: Characterization of the humoral immune response to the EBV proteome in extranodal NK/T-cell lymphoma
Source: Sci Rep. 2021 Dec 8;11:23664. doi: 10.1038/s41598-021-02788-w (PMC8655014; doi:10.1038/s41598-021-02788-w)
Supplement: Supplementary file 1 — Supplementary Information 1. [file 41598_2021_2788_MOESM1_ESM.docx]

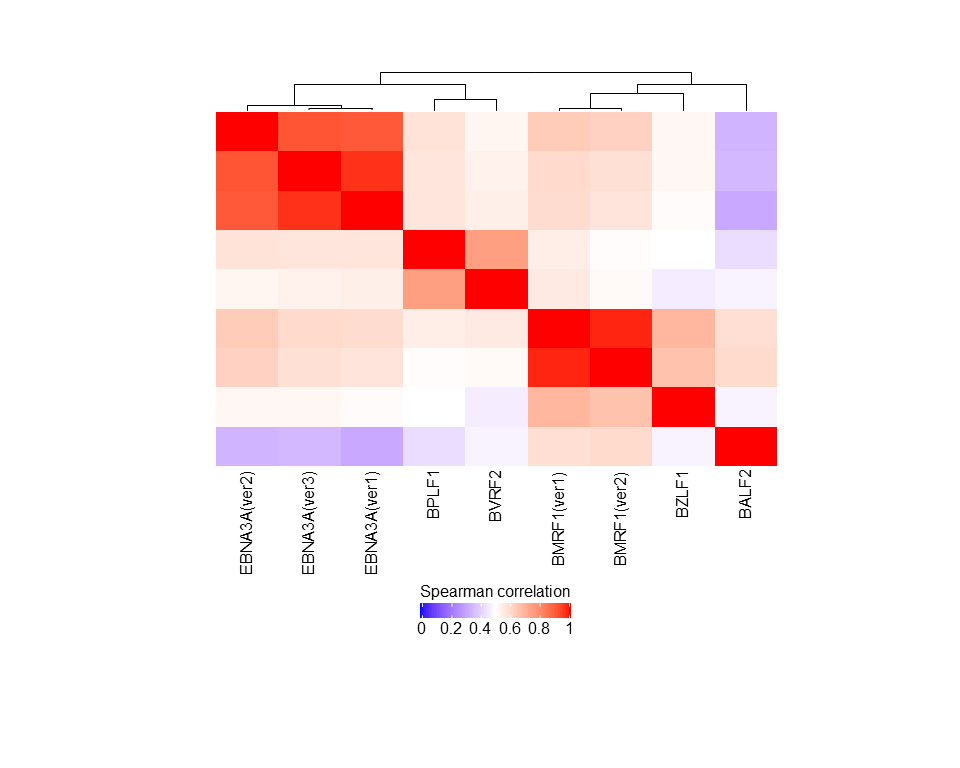


**Supplementary Figure 1.** Spearman correlation between the average immunoglobulin G (IgG) antibody responses for the nine anti-EBV IgG antibodies presented in Table 2.


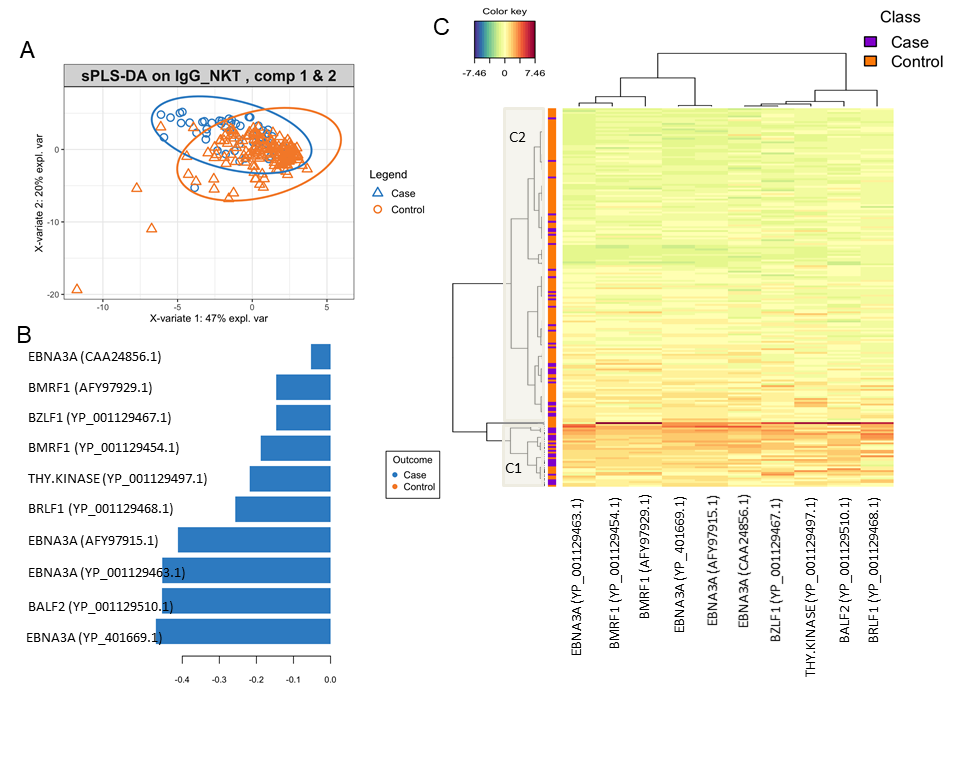


**Supplementary Figure 2**. Supervised analysis with sPLS-DA identified EBV antigens driving the discrimination between NK/T-cell lymphoma (NKTCL) cases and control . A) Sample plot depicting the first two sPLS-DA component with 95% confidence level ellipse plot; B) pyramid barplot display the top 10 EBV antigens selected on the first component of the sPLS-DA model. The length of the bar represents the importance of each antigen on the first component (importance from the bottom to top) in discriminating cases and controls. Color indicate the class type (cases- controls) where the mean of the standardize signal intensity of the antigen is maximal. C) heatmaps of the 10 discriminant features identified by sPLS-DA with both rows and columns ordered using hierarchical (average linkage) clustering shows cluster C1 enriched in NKT cases and cluster C2 enriched in controls.


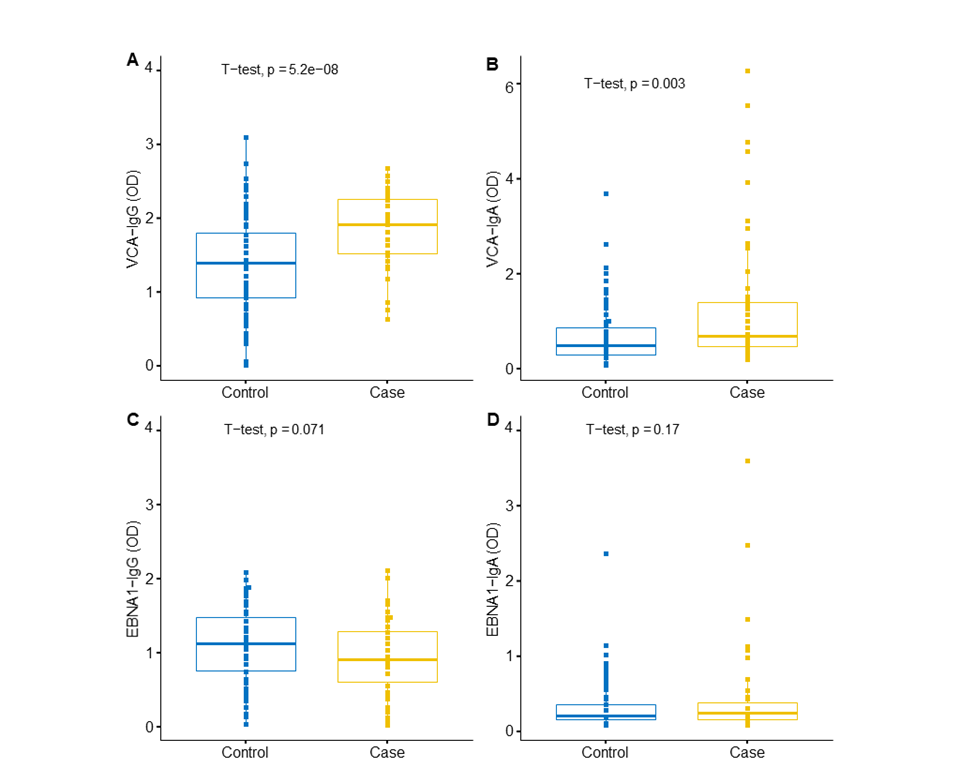


**Supplementary Figure 3.** Signal intensity for the four anti-EBV IgG antibodies tested by ELISA kits between NK/T-cell lymphoma (NKTCL) and controls, for A) VCA-IgG, B) VCA-IgA, C) EBNA1-IgG and D) EBNA1-IgA. P values from the t-test are listed.
